# Cellular prognostic markers in hepatitis-related hepatocellular carcinoma

**DOI:** 10.1186/s13027-018-0183-8

**Published:** 2018-03-27

**Authors:** A. Petrizzo, A. Mauriello, M. L. Tornesello, F. M. Buonaguro, M. Tagliamonte, L. Buonaguro

**Affiliations:** 1 Laboratory of Cancer Immunoregulation, Department of Experimental Oncology, Istituto Nazionale per lo Studio e la Cura dei Tumori, “Fondazione Pascale” – IRCCS, Via Mariano Semmola, 1, 80131 Naples, Italy; 2 Lab Molecular Biology and Viral Oncology, Department of Experimental Oncology, Istituto Nazionale per lo Studio e la Cura dei Tumori, “Fondazione Pascale” – IRCCS, Via Mariano Semmola, 1, 80131 Naples, Italy

**Keywords:** Hepatocellular carcinoma, Cellular prognostic markers, Immunoscore

## Abstract

Hepatocellular carcinoma (HCC) is the most common primary liver malignancy and accounts for about 6% of all new cancers diagnosed worldwide. Moreover, it is the third and the fifth leading cause of death from cancer in men and women, respectively. HBV and HCV chronic infection is the main risk factor for HCC.

A range of therapies are used in the management of HCC according to the extent and severity of liver disease. In this perspective, evaluation of prognosis represents a crucial step for proper management of HCC patients. However, the clinical outcome can be significantly different in HCC patients within the same stage of disease. Therefore, many efforts have been made to define new parameters with more precise prognostic value, and the search for HCC prognostic markers is gaining momentum. The present review aims at providing an update on cellular prognostic markers for HCC.

## Background

Hepatocellular carcinoma (HCC) is the most common primary liver malignancy and accounts for about 6% of all new cancers diagnosed worldwide. Moreover, it is the third and the fifth leading cause of death from cancer in men and women, respectively. The main risk factor for HCC is HBV and HCV chronic infection, accounting for an estimated 78% of global HCC cases.

A range of therapies are used in the management of HCC according to the extent and severity of liver disease, nevertheless the overall prognosis is poor and the overall 5-year survival rate is approximately 5–6% [1, 2] . HCC prognosis is closely related to its stage. However, the clinical outcome (i.e., relapse-free survival and overall survival) can be significantly different in HCC patients within the same stage of disease. Therefore, many efforts have been made to define new parameters with prognostic value in a setting of extreme heterogeneity.

Interestingly, the HCC microenvironment comprises a network of cells that play a critical role in tumor progression [3]. Indeed, several studies have shown a correlation between HCC prognosis and tumor-infiltrating immune cells. In this scenario, the prognostic value of the Immunoscore is gaining momentum and it has been the focus of several studies in the last years [4, 5].

## HCC favorable microenvironment

The healthy liver contains a large set of resident immune cells responsible for maintaining liver homeostasis through “well-balanced” inflammatory mechanisms. Despite the liver inherent tolerogenicity, resident hepatic immune cells can induce robust pro-inflammatory responses upon viral infection. Excessive inflammatory activity may turn the “well-balanced” inflammation into a dysregulated one, leading to the pathology associated with the virus infection and subsequent malignant disease. Indeed, a complex balance between inflammatory and immunoregulatory mechanisms is required to maintain local homeostasis, as well as to drive inflammation for protection against virus infection.

**Fig. 1:**
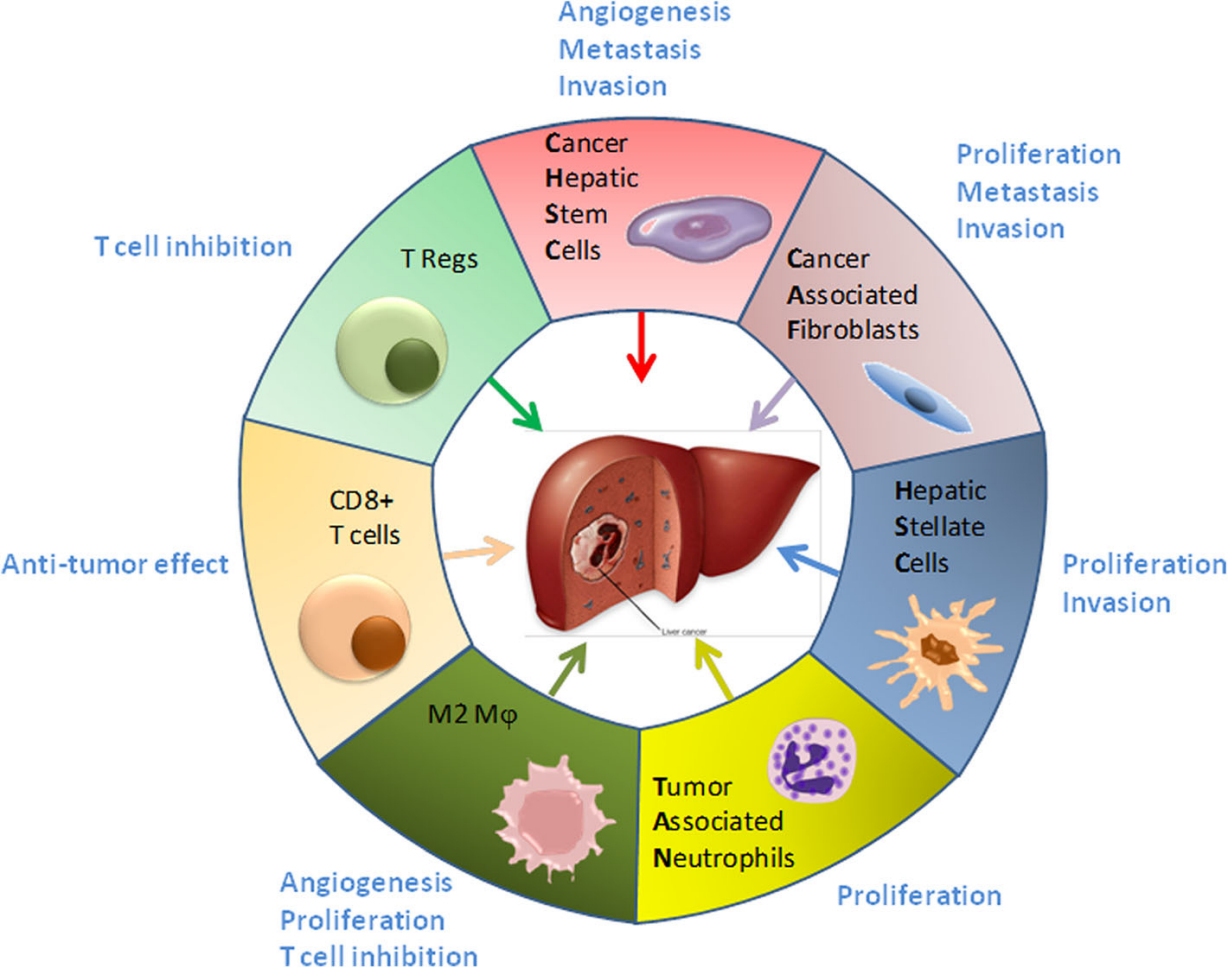
Representation of cell types infiltrating liver tumor and their effects. Each cell type found in liver cancer has been indicated with list of positive and negative effects on tumor evolution

In such a peculiar microenvironment where inflammation is responsible for both normal liver homeostasis and function, and for liver pathology, several immune cells as well as non-hematopoietic cells have shown to correlate with HCC progression. Among them, the following are worth to mention: tumor associated macrophages (TAMs), hepatic stellate cells (HSCs), cancer-associated fibroblasts (CAFs), neutrophils, cancer stem-like cells (CSLCs) and regulatory T cells (Tregs) (Fig. 1).

Macrophages represent the major component of the infiltrate and their role in tumor initiation, as well as progression has been extensively studied [6]. Macrophages can be divided into M1 (or classically activated) and M2 (or alternatively activated) [7]. M1 macrophages are mostly involved in antitumor immunity. Whereas, M2 macrophages show pro-tumorigenic effects. Accordingly, intra-tumor M2 macrophages promote tumor progression, associating with poor prognosis. In particular, peritumoral macrophage density has been shown to associate with high incidence of intra-hepatic metastasis, poor overall survival (OS) and disease-free survival (DFS) in resected HCC patients [8].

Hepatic stellate cells (HSCs) are stromal cells representing almost 30% of non-parenchymal cells in the liver [9]. Hepatic injuries induce HSCs activation and proliferation with production of extracellular matrix (ECM) and subsequent liver fibrosis [10]. Activated HSCs provide several growth factors and cytokines that play a relevant role in HCC development influencing tumor cell survival and differentiation [10, 11]. Among them, transforming growth factor-α and -β (TGF-α, TGF-β), hepatocyte growth factor (HGF), Platelet derived growth factor (PDGF), and vascular endothelial growth factor (VEGF) have been shown to establish a micro-environment which is favourable to tumor cell growth, migration and invasion [12].

Cancer-associated fibroblasts (CAFs) are major components of the tumor microenvironment [13, 14]. Their role in HCC is not fully understood, however they seem to create a favourable tumor environment by re-modulation of NK cells to an inactive phenotype with reduced anti-tumor activity [15]. More recently, it has been shown that CAFs intra-tumoral density is directly correlated with tumor size in HCC, suggesting a relevant role for CAFs in HCC pathogenesis [16].

Accumulating evidence shows that the presence of tumor-associated neutrophils (TAN) in renal cell carcinomas or in colorectal carcinomas represents a poor prognostic factor [17, 18]. Interestingly, low tumor-associated neutrophils correlate with prolonged 5-year DFS and OS also in HCC patients [19] .

Cancer stem cells (CSCs) are considered the cancer-initiating cells, responsible for tumor generation [20]. Patients with a high CSC profile show early tumor recurrence and a very poor prognosis after surgical resection of HCC [21].

Finally, among tumor-infiltrating cells, Tregs represents a marker of poor prognosis in a variety of cancers, including ovarian, breast, non-small cell lung, as well as hepatocellular carcinoma [22, 23].

Unfortunately, none of the cells described is validated for routine prognostic assessment. A better understanding of their role and clearer insight into the molecular mechanisms that are responsible for their accumulation and survival within tumors is of key importance. It is, for instance, recognized that the balance between regulatory and effector T cells plays a crucial role in determining disease outcome. In line with this evidence, the prognostic value of the tumor-infiltrating immune cells is gaining momentum and it has been the focus of several studies in the last years [24–26].

## Immunoscore as prognostic tool for HCC

Several therapeutic strategies are employed in the management of HCC according to its stage, and several staging systems are employed to estimate life expectancy of HCC patients. Among them, the TNM classification system [27].

Nevertheless, the TNM stage classification provides limited information on the outcome of patients. Indeed, patients with comparable tumor stages may experience variable clinical outcomes. In this scenario, the innovative study of Galon’s group led to the validation of the tumor-infiltrating immune cells as prognostic marker for the treatment of CRC [28]. The type, density and location of immune cells within distinct tumor regions, including tumor interior (TI) and invasive margin (IM), referred to as “Immunoscore”, was recognized as a better predictor of clinical outcome compared to standard TNM stage classification [29, 30]. In line with such evidence, several studies have been performed as attempt to translate the prognostic role of the Immunoscore (IS) to HCC [4].

The first attempt was done by Sun et al. in 2015, in a study describing the predictive value of centre tumor CD8^+^ T cells in patients with HCC [5]. The study involved two independent cohorts of HCC patients. Cohort 1 included tumor samples from 90 patients who underwent curative resection, whereas cohort 2 included tumor samples from 359 untreated HCC patients, among them, one patient was classified as HCV positive, six were classified as HCV and HBV positive, 314 patients were classified as HBV positive.

Densities of CD8^+^ and CD3^+^ T cells were evaluated in peritumoral tissue (PT) and centre tumor (CT) regions. The higher densities of CD3^+^ or CD8^+^ T cells in both regions were significantly associated with longer DFS and OS. On the contrary, neither BCLC staging nor HBV/HCV infection showed statistical prognostic impact on DFS and OS of patients.

In addition, density of CD3^+^ and CD8^+^T cells in CT and IM regions were combined into a IS scoring method, as previously established in CRC. Their results showed that patients with lower IS were significantly associated with poor prognosis, although the best association was observed with the single CD8 density in CT. Indeed, patients with CD8 CT densities > 93 cells/mm^2^ experienced significantly longer survival compared with patients with CD8 CT densities < 93 cells/mm^2^.

**Fig. 2:**
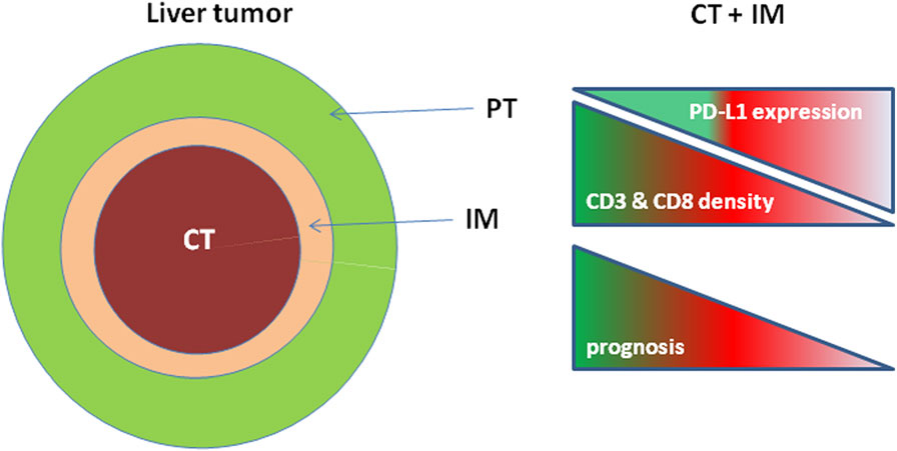
Representation of “immunoscore” applied to liver cancer. Tumor areas have been schematically indicated together with the correlation between density of T cells, PD-L1 expression in CT as well as IM and tumor prognosis

The study by Gabrielson et al. analyzed the cumulative role of intratumoral CD3^+^ and CD8^+^ T cells in combination with programmed death ligand 1 (PD-L1) as prognostic markers for HCC on 65 HCC patients (stage I to IV), who underwent primary tumor resection [4] (Fig. 2). Among them, 21 patients were HCV positive, 8 were HCV and HBV positive, 11 were HBV positive.

Immunohistochemistry (IHC) staining was performed on different areas of the tumor and surrounding liver tissues and the mean density of immune cells was used to stratify patients into groups according to the IS as defined by Galon et al. in CRC. The densities of CD3^+^ and CD8^+^ among patients with or without HBV and/or HCV infection were not significantly different.

Results confirmed a statistically significant association between intratumoral CD3^+^ and CD8^+^ T cells and frequency of HCC recurrence.

In particular, a high density of CD3^+^ immune infiltrates in the TI and IM regions correlated with recurrence only in 15% of cases compared with a 44% recurrence in patients with a low CD3^+^ cell density (*p* = 0.027). Similarly, a high density of CD8^+^ immune infiltrates correlated with recurrence of HCC in 15% of cases compared with 45% recurrence in patients with a low CD8^+^ T cell density (*p* = 0.014). In addition, high densities of CD3^+^ and CD8^+^ T cells in both TI and IM regions, along with corresponding immunoscore, were significantly associated with a prolonged RFS (*p* = 0.002).

Furthermore, expression of PD-L1 was correlated with density of CD3^+^ and CD8^+^ T cells. PD-L1 expression predicted lower recurrence rate (*p* = 0.034), as well as prolonged RFS (*p* = 0.029). Taken together, these data underline the relevance of the IS and PD-L1 expression as prognostic markers in HCC. 

The most recent study on the prognostic role of the IS in HCC was conducted by Yao et al. on a cohort of 92 patients with pathologically confirmed HBV-related primary HCC who underwent curative resection [31]. Densities of CD3^+^, CD8^+^, and CD45RO^+^ cells have been assessed in CT and IM tumor regions. Patients were stratified into five IS groups based on the combination of densities of two types of immune cells (i.e.; CD8^+^/CD45RO^+^, CD3^+^/CD8^+^ and CD3^+^/CD45RO^+^), in CT and IM tumor regions. The authors showed that a lower IS was significantly associated with poor prognosis, whereas patients with higher IS were significantly associated with longer OS. In particular, the prediction value of the combination of CD3^+^/CD8^+^ or CD8^+^/CD45RO^+^ cells resulted more significant than the combination of CD3^+^/CD45RO^+^ cells.

## Conclusions

The identification of cellular prognostic markers for hepatocellular carcinoma still represents an unmet goal. However, the studies described in the present review summarize recent evidence in support of the relevance of IS as predictive marker in patients with HCC regardless of etiology. In particular, the study performed by Gabrielson and colleagues not only supports the application of the IS as prognostic marker for HCC, but also sheds light on a complex topic that is the rationale of using PD-L1 expression as marker of prognostic significance in HCC. Moreover, the recent study performed by Yao and colleagues confirms the predictive role of the IS in patients with HBV-related HCC.

In this perspective, confirmation studies on large multi-center clinical scale would provide a significant validation of the predictive role of the IS, also in HCC, as recently observed for different tumors.

## Abbreviations

CAFs: Cancer-associated fibroblasts (CAFs)
CRC: Colorectal carcinoma
CSCLCs: Cancer stem-like cells
CSCs: Cancer stem cells
CT: Centre tumor
DFS: Disease-free survival
ECM: Extracellular matrix
HBV: Hepatitis B virus
HCC: Hepatocellular carcinoma
HCV: Hepatitis C virus
HGF: Hepatocyte growth factor
HSCs: Hepatic stellate cells (HSCs)
IHC: IMMUNOHISTOCHEMISTRY
IM: Invasive margin
IS: Immunoscore
OS: Overall survival
PDGF: PLATELET derived growth factor
PD-L1: Programmed death ligand 1
PT: Peritumoral
RFS: Relapse free survival
TAMs: Tumor associated macrophages
TAN: Tumor-associated neutrophils
TGF-α and TGF-β: Transforming growth factor-α and –β
TI: Tumor interior
Tregs: Regulatory T cells
VEGF: Vascular endothelial growth factor
